# Hydrogel Biomaterials for Stem Cell Microencapsulation

**DOI:** 10.3390/polym10090997

**Published:** 2018-09-06

**Authors:** Goeun Choe, Junha Park, Hansoo Park, Jae Young Lee

**Affiliations:** 1 School of Materials Science and Engineering, Gwangju Institute of Science and Technology (GIST), Gwangju 61005, Korea; ch70005@gist.ac.kr (G.C.); jpjh0451@gist.ac.kr (J.P.); 2 School of Integrative Engineering, Chung-Ang University, Seoul 06974, Korea; heyshoo@cau.ac.kr; 3 Department of Biomedical Science and Engineering, Gwangju Institute of Science and Technology (GIST), Gwangju 61005, Korea

**Keywords:** hydrogel, stem cell, microencapsulation, tissue engineering

## Abstract

Stem cell transplantation has been recognized as a promising strategy to induce the regeneration of injured and diseased tissues and sustain therapeutic molecules for prolonged periods in vivo. However, stem cell-based therapy is often ineffective due to low survival, poor engraftment, and a lack of site-specificity. Hydrogels can offer several advantages as cell delivery vehicles, including cell stabilization and the provision of tissue-like environments with specific cellular signals; however, the administration of bulk hydrogels is still not appropriate to obtain safe and effective outcomes. Hence, stem cell encapsulation in uniform micro-sized hydrogels and their transplantation in vivo have recently garnered great attention for minimally invasive administration and the enhancement of therapeutic activities of the transplanted stem cells. Several important methods for stem cell microencapsulation are described in this review. In addition, various natural and synthetic polymers, which have been employed for the microencapsulation of stem cells, are reviewed in this article.

## 1. Stem Cell and Stem Cell-Based Therapy

Stem cell-based therapy has recently offered new opportunities in clinical applications for conditions that are not effectively cured by conventional chemotherapy. Numerous stem cell-related studies have been performed for the purpose of treating various diseases and injuries, such as cardiovascular diseases, brain disorders, musculoskeletal defects, and osteoarthritis [1,2,3,4]. Stem cells, which possess self-renewal ability and the potential to differentiate into multiple lineages, include pluripotent stem cells (embryonic stem cells (ESCs) and induced pluripotent stem cells (iPSCs)), and multipotent stem cells (fetal stem cells, mesenchymal stem cells (MSCs), and adult stem cells) [5,6,7]. In particular, MSCs are isolated from different tissues (e.g., bone marrow, trabecular bone, adipose tissue, peripheral blood, skeletal muscle, dental pulp) and fetal tissues (e.g., placenta, amniotic fluid, umbilical cord blood, and stroma). Compared to pluripotent stem cells (i.e., ESCs and iPSCs), MSCs have a limited proliferation ability in vitro and differentiation potential. In general, stem cells give rise to various types of cells with appropriate directing cues, and eventually differentiate and integrate into host tissues in the body, which benefit the direct formation of functional tissues. Additionally, stem cells can produce various small molecules that are essential to cell survival and tissue regeneration. Substantial therapeutic efficacies of many stem cell-based therapies are attributed to such paracrine mechanisms, by enhancing angiogenesis and inducing tissue regeneration. For instance, secretory molecules from stem cells induce the proliferation and differentiation of surrounding cells and suppress fibrosis and inflammation [8,9,10]. Therefore, the sustainable release of therapeutic molecules from transplanted stem cells has been recognized as an important strategy to effectively treat various diseases.

**Figure 1:**
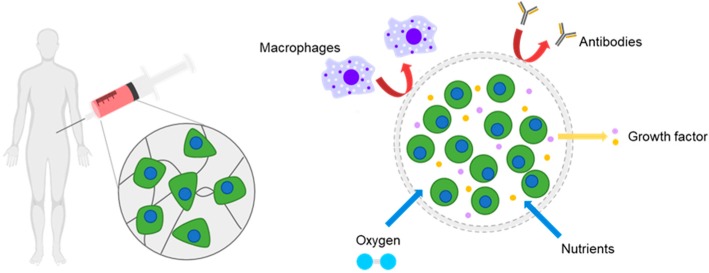
A schematic of the microencapsulation of stem cells and benefits in therapeutic applications.

Despite the considerable potentials of a stem-based therapy described above, its therapeutic efficacy is often unsatisfactory in in vivo studies. One of the reasons for this is that the transplanted stem cells lose significant viability post transplantation [11,12,13]. Injured or damaged tissues present unfavorable environments for cell growth, such as reactive oxygen species and the host’s immune responses. Also, the lack of cell-supporting signals around the transplanted stem cells leads to the eventual death of the transplanted cells. As a result, many studies have focused on stem cell transplantation with substances that can support cell survival, induce their bioactivity, and enhance cell retention at the administered sites [14,15,16]. In particular, hydrogels, which can provide tissue-like environments, have been extensively studied as delivery vehicles for stem cells. Importantly, the transplantation of stem cells in uniform micro-sized hydrogels offers convenient administration by injection in a minimally-invasive manner, allowing for patient convenience and the reduction of infection, as well as the promotion of cell viability and retention, possibly leveraging therapeutic activities of transplanted stem cells post implantation (Figure 1) [17,18]. Accordingly, many methods developed for cell microencapsulation have been recently employed for stem cell encapsulation and transplantation. Also, the properties of micro-sized hydrogels have been further tailored using proper biomaterials to obtain specific responses from stem cells for specific outcomes as stem cells sensitively respond to the properties of surrounding materials.

Cellular environments created by microgels can be engineered to encourage transplanted stem cells to exhibit multiple biological functions and thus to aid tissue regeneration by direct differentiation and/or growth factor secretion. This review specifically focuses on the microencapsulation of stem cells in hydrogels. Details of the processes of stem cell microencapsulation and associated materials are further described in the following sections.

## 2. Hydrogels

**Figure 2:**
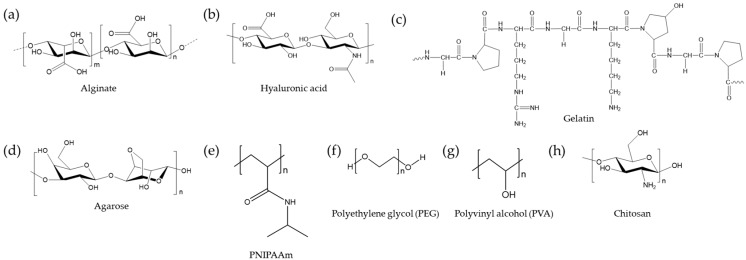
Common polymers used for hydrogels and cell delivery.

Hydrogels are crosslinked networks of hydrophilic polymers of various natural (e.g., proteins and polysaccharides) and synthetic (e.g., polyethylene glycol) polymers. Several widely used polymers for hydrogel synthesis are depicted in Figure 2. These hydrophilic polymer chains are crosslinked chemically, physically, or ionically, leading to a dramatic increase in viscoelastic properties and the maintenance of shapes and volumes in aqueous environments. In general, the hydrophilicity and softness of hydrogels make them biocompatible materials in a way that can mimic native tissues. For example, hydrogels have been widely employed in the construction of artificial extracellular matrices (ECM) to study cellular behaviors in vitro. The incubation of cells in hydrogels can serve as an efficient platform to investigate three-dimensional cell culture and its effects on stem cell growth and differentiation under various conditions. To this end, hydrogels have been extensively modified to exhibit various chemical compositions, mechanical stiffnesses, levels of degradation, and structures, which together critically affect the fates of most cells, including stem cells. For details of hydrogel and cell interactions, please see the review articles [19,20,21,22,23].

For tissue engineering applications, hydrogels provide unique advantages such as stem cell delivery materials. Hydrogels aid the retention of the encapsulated stem cells by providing biological and physical supports. Hydrogels can also serve as semi-permeable membranes with interconnected pores, which permits mass transport, including nutrient provision and waste removal from the encapsulated cells. Hydrogels can protect the encapsulated cells from the immune attack of host immune biosystems. Hydrogels can be further tailored to incorporate various cell-interactive moieties to facilitate stem cell-based therapy by promoting cell viability and/or specifically directing stem cell differentiation to target tissues [24,25]. In particular, the transplantation of micro-sized hydrogels allows for convenient injection compared to the uses of bulk hydrogels. Furthermore, micro-sized hydrogels have a high surface area and therefore permit the efficient mass transfer of oxygen and nutrients. This appropriate mass transport typically can reduce nutrient depletion causing cell necrosis, which is frequently observed when cells are encapsulated in bulk hydrogels [26,27]. Therefore, micro-sized hydrogels represent promising and effective materials for tissue engineering and cell/drug delivery applications, including stem cell delivery.

## 3. Microencapsulation

Microencapsulation indicates the process of embedding cells in micro-sized hydrogels. Microgels of tens to hundreds of microns enable the direct injection of the stem cell-embedded microgels to the targeted tissue though a needle. As cells can be administered precisely in a minimally invasive fashion, operation and patient convenience can be greatly improved. Also, the delivery of cells via microgels can enhance cell retention at the local sites, at which they usually remain without cell uptake or entering the circulatory system in the body. Original attempts to encapsulate and deliver cells in microgels were made for allogenic or xenogenic beta pancreatic islet cell transplantation for diabetic patients [28,29,30,31]. The cells in non-degrading hydrogels produced and secreted insulin while being protected from the host immune system. This concept has been subsequently employed for stem cell encapsulation and transplantation for growth factor production in vivo. Concurrently, stem cell encapsulation in a degrading hydrogel matrix and their induction to direct differentiation to functional tissues is still an effective approach for stem cell-based therapy. The production of small and uniform microgels in a biocompatible manner is, unfortunately, a challenge; hence, numerous studies have focused on the formation of small and uniform microgels with stem cells, as well as their applications in tissue engineering and drug delivery fields. Stem cell microencapsulation should utilize biocompatible and crosslinkable polymers and reliable production bioprocesses. For example, microgels must be firmly crosslinked to retain their shape and characteristics for a pre-determined time period in vivo; however, most polymers do not possess proper functional groups for a crosslinking reaction. Thus, polymers displaying crosslinkable functional groups have to be selected. Otherwise, polymers would have to be modified to introduce crosslinkable moieties. Importantly, microencapsulation processes should be biocompatible, and not cause substantial cell damage during microencapsulation.

For the microencapsulation of cells in hydrogels, several methods have been developed, including electrospraying, microfluidic emulsion, micro-emulsion, and lithographic micro-molding. Individual methods are discussed in the following sections.

### 3.1. Electrospray

**Figure 3:**
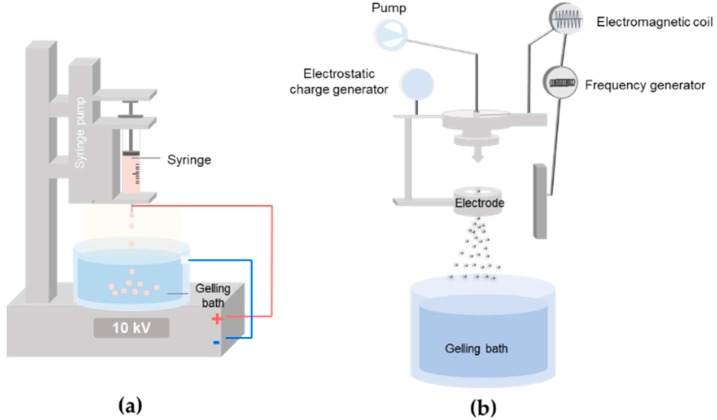
Electrospraying for microbeads production and stem cell encapsulation. Illustration of a typical electrospraying process (**a**) and a vibration nozzle-equipped system (**b**).

Electrospraying is one of the most commonly used techniques for the microencapsulation of cells. Micro-sized droplets are simply produced from a flowing solution in a high-voltage gradient, in which the force balance between electrical repulsion and tension leads to the formation and ejection of small liquid droplets (Figure 3a). Spraying conditions, such as nozzle diameter, flow rates, and applied voltages, determine the sizes and uniformity of the microbeads. The sprayed droplets containing hydrophilic polymers and cells quickly crosslink in an aqueous bath. However, a high electric field can have a negative effect on cell viability due to electrical and thermal damages [32,33]. Therefore, optimal voltages are generally employed to produce uniform small microdroplets and high cell viability. Ionically crosslinkable alginate, most widely used with electrospraying, immediately crosslinks with divalent ions (e.g., calcium and barium) once the droplets come into contact with the divalent ion-containing solution. Taking advantage of this alginate-based simple microencapsulation, various composites consisting of alginate and other biologically active polymers can be further electrosprayed together in the form of a semi-interpenetrating network (semi-IPN), where alginate provides structural stability. In addition to ionic crosslinking, electrosprayed droplets can be crosslinked by photopolymerization for gelation [32,34,35]. Cell solution containing [meth]acrylated polymers and photoinitiators is electrosprayed and UV light exposure usually follows for gel formation. It is also possible for alginate to serve as a temporary template for the production of microbeads of other polymers. For example, alginate and methacrylated hyaluronic acid (HA) have been electrosprayed into a calcium-containing bath to form uniform microspheres based on the ionic crosslinking of alginate, followed by UV photopolymerization of the [meth]acrylated HA within the microgels. Then, alginate can be removed from the microspheres by the addition of EDTA [36]. Note that UV photopolymerization is a common method for hydrogel synthesis for various cell-related studies, as it offers good temporal and spatial control over gelation at physiological temperatures and pH levels.

A vibrational technology uses a vibrating nozzle for a feeding solution to generate dispersed droplets (Figure 3b). As the vibration progresses in the Plateau-Rayleigh instability region of resonance, droplets of uniform distribution are formed. In many cases, a vibrating nozzle is equipped with electrospraying systems, in which an electrical field stabilizes the droplet jet and disperses the droplets without aggregation. The sizes of generated droplets are adjusted by changing the vibrational frequency, amplitude of vibration, solution pumping rate, and distance between the nozzle and the gelling bath [37,38].

### 3.2. Droplet-Based Microfluidics

**Figure 4:**
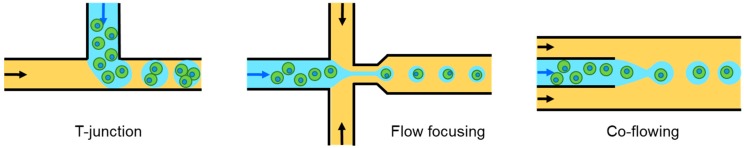
Microfluidic-based microdroplet generation by T-junction, flow focusing, and co-flowing methods.

The field of microfluidics has garnered great attention for the fabrication of miniaturized systems permitting the precise microscale control of fluids. With the unique abilities of microfluidic systems with fine valve controls, geometry of channels, and flow rate controls, droplets of several to hundreds of microns in size with a narrow size distribution, controllable shapes, and hierarchical structures can be effectively produced [39]. It is even possible to encapsulate a single cell per droplet. Droplet-based microfluidics is largely divided into two methods; active and passive. Electric, magnetic, and centrifugal methods are active methods, whereas cross-flowing, flow focusing, and co-flowing methods are passive methods [40,41]. Active methods introduce a way of changing interfacial instability by modifying the force balance at the droplet interface by supplying additional energy through external elements. These are frequently used in extreme situations such as a highly viscous solution or a deterministic cell number [42,43]. Passive droplet-based microfluidics use two immiscible continuous phases in which aqueous droplets containing cells are formed in the dispersed phase by flow shearing (Figure 4). For details on these active and passive droplet-based microfluidics methods, please see the recent review article [40]. Of the two methods, the passive method is commonly used to encapsulate cells. Cells are mixed in an aqueous solution containing hydrophilic pre-polymers. The cell-laden solution is subjected to shearing off by a water-immiscible continuous fluid (e.g., oil phase), resulting in the formation of dispersed particles with well-defined shapes and sizes in microfluidic devices. Typically, microfluidic devices with a ‘T’ junction, flow-focusing, or co-flowing components are used to form aqueous droplets in the oil phase (Figure 4). However, the use of oil can reduce cell viability because nutrients may not be sufficiently supplied at the surface contacting the oil phase during microbead production in microfluidic devices. The produced droplets are usually gelled in a microfluidic device. For example, cell-laden alginate droplets are ionically crosslinked by contact with a calcium-containing continuous oil phase. Agarose-based droplets undergo solidification by lowering the temperature. In addition, UV radiation follows cell-containing microdroplets for in situ photocrosslinking.

### 3.3. Photolithography/Micro-Molding

**Figure 5:**
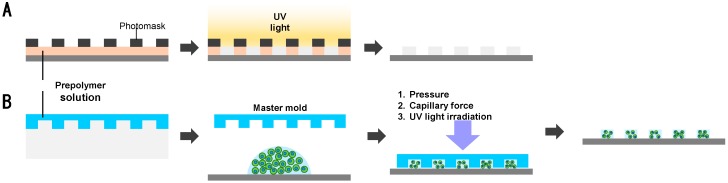
Schematic illustration of (**A**) photolithography and (**B**) micro-molding methods for cell encapsulation in microgels.

Micro-molding/photolithography utilizes lithographic techniques to form, cure, and release cell-containing microgels in microarrays [24,25,26]. A photolithographic method uses a patterned photomask that has various micro-sized shapes in array. UV radiation through the photomask leads to photopolymerization according to the mask patterns (Figure 5A). Then, the unexposed gel/cells are simply removed by washing as non-crosslinked pre-polymers that are easily dissolved out and the polymerized cell-laden microgels are retrieved. Various photopolymerizable polymers with various cells have been used to produce cell encapsulation in various shapes. For example, beads, sheets, and fiber hydrogels that embed cells can be produced with different photomasks. In addition, micro-molding is a technique that loads cell-containing pre-polymer solutions in the microholes, followed by gelation and release by gentle shaking. The production of arbitrary shapes and can be simply obtained and mass produced. For micro-molding, stimulus responsive hydrogels and UV photopolymerization are usually employed. In the case of temperature-sensitive polymers, they exhibit sol-gel transition at a certain temperature. For example, poly(*N*-isopropylacrylamide) (PNIPAAm) or Pluronics^®^ form a physical gel above the lower critical solution temperature (LCST). A solution of these polymers and cells is put in a mold at a temperature below the LCST, and allowed to gel by increasing the temperature to above the LCST. In the case of photopolymerization, a solution containing pre-polymer, photo-initiators, and cells is transferred onto the surface of patterned molds. After UV irradiation for crosslinking, fabricated gels are retrieved. Ionic gelation after micro-molding is also possible. Lee et al. prepared cell spheroids in alginate using an array of concaved wells. They crosslinked cell-containing alginate in wells by diffusing calcium ions from the porous membranes on the top of the micro-molds [44]. With complicated structures fabricated by the micro-molding method, shape-based assemblies of microgels can be further realized. Du et al. fabricated cross- and rod-shaped microgels and successfully demonstrated their selective assembly [45]. However, these methods have several drawbacks. As the aspect ratio of the mold increases, the feature quality of fabricated hydrogels dramatically decreases. Also, several factors (drying during processing, temperature change, UV initiators in UV crosslinking, and light intensity) have to be carefully considered as these have detrimental effects on cell viability. For further details on micro-molding techniques for cell encapsulation, please see the recent reviews [46].

### 3.4. Emulsification

**Figure 6:**
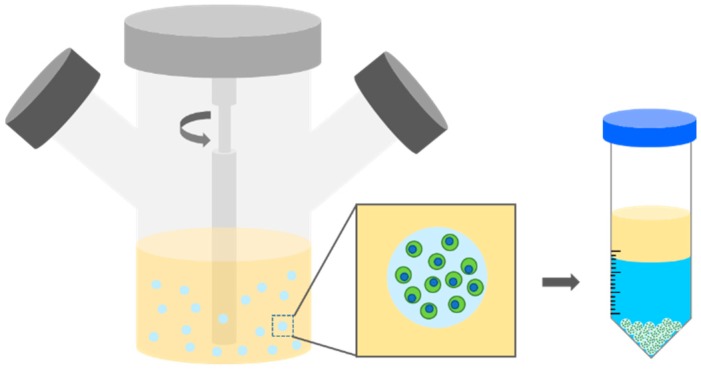
Stem cell encapsulation by an emulsion method.

Cell embedded polymer solution droplets are formed conventionally in an oil phase via a water-in-oil emulsion (Figure 6) [36,47,48,49]. Hoesli et al. created a water-in-oil emulsion by agitating alginate solution with CaCO_3_ in mineral oil. Then, internal gelation occurred to form microgels during and after emulsion. Emulsified hydrogels were easily collected from the oil phase by centrifugation. Emulsification possesses several important advantages for cell encapsulation, including low equipment cost, low production cost, and scalability. However, this method has raised concerns, such as broad size distribution and cell damage at the oil interfaces.

This section basically provides methods to form hydrogel microbeads. Conditions for individual methods should be carefully optimized to produce well-defined gels while maintaining stem cell viability. For example, the uses of hydrophobic substances during droplet microfluidics and micro-emulsion processes can potentially affect stem cell viability. Importantly, finding scalable and biocompatible processes represents a great challenge for clinical applications of stem cell microencapsulation.

## 4. Biomaterials for Microencapsulation

Materials for stem cell microencapsulation for clinical applications should meet two important criteria; processability and bioactivity. As mentioned above, micro-sized gels have to be cured (crosslinked) to maintain their shapes/structures and ensure the residence of the administered cells at the injected tissues. Only hydrogel materials possessing crosslinkable groups can be used. If not, polymeric chains are chemically modified to introduce crosslinkable groups. Typical bulk chemical crosslinking agents are often toxic to stem cells. Therefore, ionic crosslinking, photopolymerization, and mild chemical reactions are employed for crosslinking and cell encapsulation. Another important aspect is that hydrogels have to be biocompatible and preferentially bioactive to support the growth and differentiation of the encapsulated stem cells according to their intended purposes. For tissue engineering, degradable hydrogels are preferred to allow the encapsulated cells and host tissues to newly fill the space and integrate with each other after the matrix hydrogel degrades. In these cases, the design of degradation rates and mechanisms (e.g., enzymatic or hydrolytic) are essential. On the other hand, for drug delivery purposes (based on secretomes from the encapsulated stem cells), non-degradable (or long-lasting) materials are desirable to ensure the production of therapeutic molecules for sufficiently long periods without severe decomposition under exposure to host immune systems. The biocompatibility and bioactivity of encapsulating materials are critically associated with cell viability. When cells are not bound to the ECM properly, cells are known to undergo apoptosis, called “anoikis”. In particular, tissue environments post injection are usually harsh. Injection processes themselves cause the disruption of blood vessels, releasing inflammatory cytokines and reactive oxygen species. Therefore, cell-supporting moieties from hydrogel materials, which lead to cell-matrix binding, are highly desirable to promote cell viability by anti-apoptotic signal transduction. The binding of the ECM to cell surface receptors (i.e., integrin) forms a focal adhesion complex and activates focal adhesion kinase (FAK). FAK activates its downstream (e.g., ERK, MAPK, PI3-K, and AKT) signaling and affects cell adhesion, proliferation, and survival [50,51,52]. In addition, the incorporation of specific polymers or molecular units that can direct cell differentiation to specific lineages is desirable to form functional tissues [53].

### 4.1. Alginate and Alginate Derivatives

Alginate is obtained from brown algae and consists of guluronic acid and mannuronic acid. Alginate readily crosslinks with divalent cations, such as calcium and barium. Numerous alginate-based biomaterials have been widely used for biomedical applications because of their biocompatibility and ease in crosslinking. Alginate has been the most used polymer matrix for microbeads production and cell encapsulation as its gelation occurs quickly under very mild conditions without using organic solvent nor toxic chemicals in gelation [54,55,56]. Alginate and alginate derivatives have been widely used for stem cell encapsulation with various methods (Table 1). Penolazzi et al. encapsulated Wharton’s jelly (WJ)-derived MSCs in alginate microbeads (550–650 µm in diameter) by a vibrational nozzle technique. They found that the encapsulated WJMSCs produced secretomes (e.g., interleukins, chemokines, growth factors, and soluble forms of adhesive molecules) [37]. Alginate microgels are often coated with polycationic polymers, such as poly-l-lysine (PLL), to enhance the membrane integrity [57]. Although alginate has been fundamentally easy to use for stem cell microencapsulation, alginate does not present biologically active moieties. Hence, alginate has been modified or composited with other biologically active materials to improve cellular interactions and thus the efficacy of stem cell-based therapy. Composites of alginate with other biologically active molecules (e.g., hyaluronic acid and gelatin) are simply prepared for various microencapsulation methods to the extent that the addition of other substances to alginate does not hinder ionic crosslinking and hydrogel integrity. Yao et al. electrosprayed a composite solution of alginate and gelatin with MSCs and found that the adipogenic differentiation of the encapsulated MSCs was enhanced in gelatin-introduced microbeads compared to those in alginate microbeads [58]. Hernandez et al. formulated alginate with HA for MSC encapsulation and found that at the optimal compositions (1% alginate and 0.25% HA), both cell growth and the release of therapeutic proteins were greatly promoted [59]. Another strategy to imbue alginate with bioactivity includes the covalent grafting of alginate with ECM-derived peptides. These efforts allow alginate-based microbeads to better mimic the natural ECM environments that provide multiple specific signals to cells. For example, the incorporation of RGD (arginine-glycine-aspartic acid) peptides, derived from vitronectin and fibronectin, to alginate substantially stimulates stem cell viability, ability to produce therapeutic proteins, and functional differentiation. Note that numerous biologically active peptides have been identified and thus tethering such peptides would be applicable to stem cell encapsulation. Still, the effects on stem cells need to be carefully examined. Moreover, non-degrading alginate can be made degradable. The partial oxidation of alginate leads to the hydrolytic degradation of alginate in vitro as well as in vivo, in which oxidation degrees can be further controlled to modulate the degradation rates.

### 4.2. Hyaluronic Acid and Its Derivatives

HA is a linear polyanionic polysaccharide consisting of *N*-acetyl-glucosamine and glucuronic acid. HA is a major ECM component existing in the human body, mostly serving as a tissue-filling component in connective tissues. In addition, HA exhibits several important biological characteristics associated with angiogenesis, development, and anti-inflammatory activity [67,68]. In the material aspect, HA does not possess crosslinkable moieties, and thus it is chemically modified with various groups (e.g., methacrylate, adipic acid dihydrazide, tyramide, benzyl ester, hydrazide, and thiol groups) for crosslinking [68,69,70]. These chemical groups form crosslinks via photopolymerization or chemical coupling reactions (e.g., Michael-type additions). Examples of hyaluronic acid-based stem cell microencapsulation are shown in Table 2. Khademhosseini et al. demonstrated cell-laden HA microgels by a micro-molding technique. They placed a mixture of methacrylated HA, photoinitiators, and ESCs on a patterned PDMS (polydimethylsiloxane) master mold, followed by UV exposure for curing. They reported that photoinitiator concentrations and UV light intensity were important to cell viability (this report showed 82% cell viability) [71]. Khetan et al. synthesized HA modified with methacrylate and maleimide, with which they first formed hydrogels using thiol-containing cell-adhesive oligopeptides via a Michael-type reaction. Methacrylate groups in HA were subsequently used for photopolymerization. The properties of hydrogel and cell spreading were varied to give optimal direction to the differentiation of the encapsulated MSCs with high cell viability [72]. Bae et al. produced HA microbeads using alginate micro-templates. They extruded alginate, methacrylated HA, *N*-vinylpyrrolidone, and photoinitiators into a calcium-containing bath to ionically crosslink microbeads. Then, they photopolymerized the microbeads and subsequently extracted alginate from the beads by EDTA treatment [36]. Chung et al. microencapsulated MSCs in HA by the micro-molding and photopolymerization of methacrylated HA. They observed enhanced chondrogenic differentiation from the encapsulated MSCs even in the absence of TGF-β3, presumably due to the interaction between HA and CD44 receptors on MSCs [67]. Several efforts were made to encapsulate cells in HA microgels without initiators using spontaneous chemical reactions. For example, Guermani et al. produced microbeads with micro-printing using thiolated HA (HA-SH) and methacrylated gelatin (GelMA), which reacted with each other via a thiol-ene reaction under a physiological condition [73]. In order to retrieve cells and cultivate a large number of cells, a thermosensitive property was introduced to HA by incorporating thermoresponsive polymers. Ekerdt et al. polymerized a temperature-responsive polymer of vinyl sulfone-modified HA (HA-VS) and thiolated PNIPAAm (PNIPAAm-SH) by a micro-molding or emulsion method. Through sol-gel transition, the cells encapsulated in HA/PNIPAAm hydrogels could be easily retrieved by lowering the temperature to 4 °C [47].

### 4.3. Gelatin and Gelatin Derivatives

Gelatin is derived by the irreversible hydrolysis of collagen. Gelatin is composed of repeated Gly-X-Y amino acid sequences, where X is usually proline and Y is hydroxyproline. Gelatin offers several beneficial properties for biomedical applications such as good biocompatibility, biodegradability, and non-immunogenicity. Gelatin supports the attachment and proliferation of various types of cells, including stem cells. According to the temperature change, gelatin undergoes phase transitions. At body temperature, it remains in a solution state, but at lower temperatures it turns to a gel-like state. As the result, pristine gelatin is not in a gel state at body temperature. Hence, gelatin is frequently modified to crosslink to ensure a stable gel state at body temperature. Based on these properties, gelatin-based materials have been used for stem cell microencapsulation (Table 3). Nichol et al. fabricated methacrylated gelatin (GelMA) microgels using micro-patterning and photopolymerization. Encapsulated cells displayed well elongated shapes and good proliferation [75]. Physical properties of gelatin-based hydrogels could be further improved by combinations with other macromolecules, such as gellan gum [29] or polyethylene diacrylate (PEGDA) [48].

### 4.4. Agarose

Agarose is composed of 1,4-linked 3,6-anhydro-α-1-galactose and 1,3-linked β-d-galactose derivatives. Like gelatin, it has a thermoresponsive property (a gel state at room temperature and a solution state at an elevated temperature). This temperature-dependent phase change makes agarose a beneficial material for the easy modulation of its mechanical properties during particle synthesis. For example, cell-containing agarose solutions can be prepared and emulsified at 37 °C, which can be then gelated to microgels in an ice bath [56]. As shown in Table 4, a few studies were performed for stem cell encapsulation in agarose. Tumarkin et al. fabricated agarose microgels with microfluidics though a T-junction. They demonstrated the controlled encapsulation of two different types of cells using two independent syringe pumps. They also reported the feasibility of their system to control cell population in hydrogels and to investigate paracrine interactions among cells [30]. In addition, agarose is substituted with peptides for better cell adhesion as it exhibits the low adsorption properties for cells and proteins [80,81,82].

### 4.5. PEG and Its Derivatives

PEG is one of the most widely used synthetic biomaterials because of its hydrophilicity, biocompatibility, high flexibility, inertness, and non-biofouling property. Its synthetic and conjugation chemistry is well developed. Hence, various PEG derivatives (e.g., [meth]acrylate, allyl ether, maleimide, *N*-hydroxysuccinimide (NHS) ester, vinyl ether, and vinyl sulfone) can be easily obtained and used for various conjugation and cross-linking reactions. Furthermore, PEG can be modified to incorporate biointeractive moieties for the modulation of cell proliferation and differentiation, using peptides such as the Arg-Gly-Asp (RGD) peptide. PEG gelation is achieved by photopolymerization using [meth]acrylated PEG [45,85,86]. Yeh et al. fabricated PEGDA hydrogels using a micro-molding technique in conjunction with photopolymerization and found high cell viability at lower UV exposure times. The sizes and characteristics of PEG hydrogels could be simply modulated by varying the crosslinking density and/or combining it with other polymers possessing photopolymerizable functional groups. In particular, PEG is biologically an inert polymer, for which bioactivity or processability is often introduced by the incorporation of bioactive molecules in a well-defined chemistry. Therefore, PEG-based materials have been widely used for microencapsulation of various stem cells with various methods (Table 5). Jeon et al. methacrylated partially oxidized alginate and used it with eight-arm PEG amine to fabricate hydrogels with micro-molding. The size of microgels could be easily adjusted by the predetermined mold dimensions. They found that a larger size led to the substantially better differentiation of MSCs [87]. Siltanen et al. encapsulated ESCs in heparin/PEG microgel using a microfluidic technique. They attempted to induce the endodermal differentiation of ESCs by a controlled release of growth factors (e.g., Nodal, FGF-2, activin A) from the gels based on the strong affinity of heparin to growth factors [88]. Rossow et al. employed initiator-free gel formation using dithiolated PEG and acrylated PEG; this Michael-type addition reaction can avoid damage to cells related with toxic initiators [85]. Likewise, four-arm PEG maleimide and thiolated PEG were used for initiator-free cell encapsulation [89]. Recently, the mitigation of PEG-related issues has been studied, such as long-term immune reactions to native PEG and the in vivo stability of hydrogels. For example, amphiphilic polymer (Mal-PEG-lipid) showed reduced thrombogenicity and improved graft survival.

### 4.6. Other Polymers

In addition to the hydrogel materials discussed above, there are numerous polymers that can potentially be used for stem cell microencapsulation. Although we briefly highlight some polymers and their uses for cell microencapsulation in this review, it should be noted that some polymers have been used for the encapsulation of non-stem cells (e.g., fibroblasts, myoblasts, and hepatocytes) for the purpose of studying fundamental cell-material interactions, drug screening, or tissue engineering applications. For example, chitosan is a natural cationic polymer that is made by treating an alkaline substance to chitin and copolymer of β-(1→4)-linked d-glucosamine and *N*-acetyl-d-glucosamine. Chitosan has been widely used in tissue engineering because of its excellent biocompatibility, biodegradability, hydrophilicity, and structural similarity to glycosaminoglycans (GAGs) [62,93]. Fukuda et al. prepared hydrogels by photocrosslinkable chitosan using a micro-molding method. They encapsulated hepatocytes and fibroblast and performed a co-culture experiment to study cell-cell interactions [93]. PNIPAAm is a well-known synthetic polymer that has a thermoresponsive property. In contrast to gelatin and agarose, PNIPAAm forms a gel at a temperature higher than the LCST (~32 °C) through reversible sol-gel transition, whereas it remains in a solution state at lower temperatures due to the dominant interactions among the hydrophobic groups in PNIPAAm. Hackelbusch et al. fabricated a hydrogel of PNIPAAm grafted with poly(*N*-2-hydroxyproyl)-methacrylamide, poly(hydroxyethyl methacrylate), and cyclooctyne-functionalized PEG. This system permitted the control of physical properties by differing the length of the polymer chains and grafting density. They also reported the high viability of the cells encapsulated with microfluidic devices. Poly(vinyl alcohol) (PVA) is a non-ionic synthetic polymer, which presents excellent hydrophilicity, biocompatibility, and a low friction coefficient. A study done by Young et al. demonstrated that the encapsulation of fibroblasts in methacrylated PVA by electrospray and photopolymerization methods indicated high cell viability [34].

Various materials have been studied for the microencapsulation of (stem) cells, as described above. The choice or modification of materials has to be carefully considered depending on specific methods. Furthermore, materials capable of providing specific signals to stem cells are highly desirable, as stem cell performances (e.g., cytokine production and direct differentiation) are greatly affected by their microenvironments. Therefore, the properties of the microgels embedding stem cells should be tailored to deliver specific signals to the encapsulated stem cells.

## 5. Challenges and Future Directions

We reviewed studies on stem cell encapsulation in microgels, highlighting the related microencapsulation methods and hydrogel materials. Despite recent promising results and potentials, there are still several issues remaining. First of all, the viability of encapsulated cells is relatively low and questionable. In particular, photopolymerization with radial initiators during encapsulation often cause severe damage to cell viability. Oil emulsion is also known to damage the cell’s lipid membrane. Hence, efficient and biocompatible systems need to be established to reduce the use of radicals and light intensity or contact time with the oil phase. In addition, most of the methods are developed and tested for encapsulation at a small laboratory scale, raising uncertainty toward large-scale production while meeting the requirements of good manufacturing production (GMP) guidelines. Given that a large amount of stem cells (about 10^7^–10^10^ cells per patient) is administered in clinical trials, it is a challenge to uniformly encapsulate a large number of cells in a state that maintains cell viability or functionality during and after the culture processes. Also, advances in nano- and micro-technology and material synthesis will offer new opportunities for stem cell microencapsulation. Furthermore, in vivo studies of the effectiveness of stem cell-laden microgel delivery are required to provide clear insights on the roles of stem cells in pathological tissues and the interactions between stem cells and materials. Based on the accumulated results, processes and materials for stem cell microencapsulation will be further tailored according to their specific applications.

## 6. Conclusions

Stem cell microencapsulation in hydrogels can leverage the utilities of stem cell-based therapy for tissue engineering and drug delivery applications. The microencapsulation of stem cells and delivery has been particularly devoted to circumventing current issues related to stem cell transplantation, such as low cell viability, poor retention in vivo, lack of functional activities (secretome production or direct differentiation), and direct exposure to host immune responses. In this review, we highlighted various methods and various hydrogel materials for stem cell microencapsulation with multiple studies demonstrating the potential of stem cell encapsulation and delivery for treating various diseases. Several challenges (e.g., biocompatibility, processability, and in vivo efficacy) need to be further addressed in future studies to promote the therapeutic activity and applicability of microencapsulated stem cells.

## Figures and Tables

**Table 1:** Examples of alginate-based stem cell microencapsulation.

Materials	Methods	Stem Cell Type	Applications	Ref.
Alginate	Vibrational nozzle Vibrational nozzle Electrospray	ADMSCs WJMSCs MSCs	Retention Cell functionality Hind limb ischemia	[60] [37] [61]
Alginate/PLL	Electrospray	ESCs	Hematopoietic, neural ectoderm differentiation	[62]
Alginate-PLL-alginate	Electrospray	MSCs	Diabetes	[63]
RGD-alginate	Electrospray	MSCs	Myocardial infarction	[64]
RGD-alginate/PLL	Microfluidics	PDLSCs and GMSCs	Bone regeneration	[65]
Alginate/gelatin	Electrospray	ADMSCs	Adipogenic differentiation	[58]
Alginate/HA	Electrospray	MSCs	Viability, chondrogenic differentiation	[59]
Alginate/PEG	Microfluidics	MSCs	Liver fibrosis	[66]

ADMSCs (adipose-derived mesenchymal stem cells); WJMSCs (Wharton’s jelly-derived mesenchymal stem cells); MSCs (mesenchymal stem cells); PDLSC (periodontal ligament stem cells); GMSC (gingival mesenchymal stem cell).

**Table 2:** Examples of hyaluronic acid-based stem cell microencapsulation.

Materials	Methods	Stem Cell Type	Applications	Ref.
MAHA	Micro-molding	ESCs	Viability, retrieval	[71]
MAHA/PEGDA	Micro-molding	MSCs	Chondrogenesis	[67]
RGD-MAHA	Photopolymerization	MSCs	Osteogenic, adipogenic differentiation	[72]
HA-SH/PEGTA	Micro-drop printing	MSCs	Osteogenic differentiation	[73]
VS-HA or anti-Fas HA	Microfluidics	NSCs	Survival	[74]
HA/PNIPAAm	Emulsion	iPSCs, ESCs	Cell expansion, maintaining pluripotency	[47]

MAHA (methacrylated HA); PEGDA (PEG-diacrylate); HA-SH (thiolated HA); PEGTA (PEG-tetraacrylate); VS-HA (vinyl sulfone-modified HA).

**Table 3:** Examples of gelatin-based stem cell microencapsulation.

Materials	Methods	Stem Cell Type	Applications	Ref.
Gelatin	Emulsion	MSCs	Chondrogenic differentiation	[76]
Gelatin-Ph	Microfluidics	ADMSCs	Cell aggregates formation	[77]
Gel MA	Micro-molding	iPSCs	Cardiac differentiation	[78]
GelMA/nHA	Photolithography	PDLSC	Osteogenic differentiation, periodontal tissue regeneration	[79]

Gelatin-Ph (phenolic hydroxyl group containing gelatin); GelMA (methacrylated gelatin); nHA (nanohydroxylapatite); PDLSC (periodontal ligament stem cells).

**Table 4:** Examples of agarose-based stem cell microencapsulation.

Materials	Methods	Stem Cell Type	Applications	Ref.
Agarose	Microfluidics	ESCs	Paracrine interaction	[83]
	Microfluidics	ESCs	High-throughput generation	[84]

**Table 5:** Examples of PEG-based stem cell microencapsulation.

Materials	Methods	Stem Cell Type	Applications	Ref.
PEG-amine/oxidized methacrylate alginate	Photolithography	ADMSCs	Osteogenic, chondrogenic, adipogenic differentiation	[87]
PEGDA	Micro-molding	ESCs	Viability	[90]
PEGDA	Emulsion	MSCs	Ossification	[91]
PEGSH/PEGDA/Hep-MA	Microfluidics	ESCs	Endodermal differentiation	[88]
PEG-4MAL	Microfluidics	MSCs	Viability, functionality	[89,92]

PEGDA (PEG-diacrylate); PEGSH (PEG-thiol); Hep-MA (heparin methacrylate); PEG-4MAL (PEG-4 maleimide).
